# Infections and CAR-T cells for the treatment of lymphoid malignancies: a narrative review

**DOI:** 10.3389/fmed.2026.1830105

**Published:** 2026-06-16

**Authors:** Carolina Secreto, Filippo Fasano, Davide Stella, Mattia Novo, Barbara Botto, Marco Cerrano, Roberto Freilone, Alessandro Busca

**Affiliations:** Division of Hematology and Stem Cell Transplant Center, AOU Città della Salute e della Scienza, Turin, Italy

**Keywords:** antimicrobial prophylaxis, chimeric antigen receptor-T cell (CAR-T cell) therapy, infections, lymphoid malignancies, risk factors

## Abstract

The use of chimeric antigen receptor-T (CAR-T) cells have revolutionized the therapeutic paradigm of patients with lymphoid malignancies. However, infectious complications represent a frequent CAR-T cell-related adverse event, potentially being a major hurdle for the successful outcome of the patients. The infection incidence follows a biphasic pattern, with “early” infections rising during the first 30 days after CAR-T cells infusion, and “late” infections from day 30 onward. Overall, bacterial infections prevail in the early phase after therapy, with a switch to viral or opportunistic infections in the long-term period, while invasive fungal infections are rare events after CAR-T therapy. Risk factors associated with infectious complications include host-related factors such as the underlying malignancy and previous treatments, and treatment-related factors [CAR-T cell product, cytokine release syndrome (CRS), immune effector cell associated neurotoxicity syndrome (ICANS), immune effector cell–associated hematotoxicity (ICAHT), hypogammaglobulinemia]. Careful attention to signs and symptoms of infections is mandatory for an optimal management of patients undergoing CAR-T therapy, and strategies to mitigate infectious risk are clinically relevant: indeed, over half of non-relapse mortality in these patients is attributed to infections. In the present review we attempt to summarize the current knowledge on infectious complications occurring in patients receiving CAR-T cell therapy for lymphoid malignancies in order to provide the readers tools for better management and prevention strategies.

## Introduction

The broadening molecular landscape of a large number of hematologic malignancies have set the ground for personalized therapies in recent years. Chimeric antigen receptor-T (CAR-T) cells have revolutionized the therapeutic paradigm of patients with lymphoid malignancies (1): several anti-CD19 CAR-T cell products demonstrated impressive efficacy for the treatment of relapsed/refractory (R/R) large B-cell lymphoma (LBCL), mantle cell-lymphoma (MCL) and follicular lymphoma (FL), and received regulatory approval in these settings (2–9).

In addition to CAR-T peculiar toxicities, like cytokine release syndrome (CRS) and immune effector cell associated neurotoxicity syndrome (ICANS), infections represent a frequent CAR-T cell-related adverse event, potentially being a major hurdle for the successful outcome of the patients and representing the most frequent cause of non-relapse mortality (10, 11).

Although the duration and the depth of neutropenia, due to lymphodepleting (LD) chemotherapy or CAR-T cell–associated hematotoxicity, is traditionally considered as the most relevant factor influencing the risk of infectious complications, adaptive immune defects associated with the infusion of CAR-T cells have emerged as elements potentially leading to the development of infections, including B-cell aplasia and hypogammaglobulinemia (2, 3, 12). Furthermore, risk factors associated with infectious complications encompass host-related factors [i.e., underlying malignancy, previous treatments including hematopoietic stem cell transplantation (HSCT), previous infections], and treatment-related factors [CAR-T cell product, CRS, ICANS, use of steroids or tocilizumab, and immune effector cell-associated hemophagocytic lymphohistiocytosis-like syndrome (IEC-HS)] (Table 1).

**TABLE 1:** Risk factors associated with infections following CAR-T cell therapy.

Patient-related risk factors	Disease-related risk factors	CAR-T-related risk factors
Age	Active/ Refractory NHL	Type of CAR-T cell product
Comorbidities	Type of NHL	LD regimen
Prior treatments (including HSCT)	Disease-related neutropenia	CAR-T complications and treatments
Pre-infusion neutropenia		CRS
Previous infections		ICANS
		ICAHT
		IEC-HS
		Steroids
		Tocilizumab
		Prolonged immunosuppression
		B-cell aplasia
		Delayed T-cell recovery
		Hypogammaglobulinemia

HSCT, hematopoietic stem cell transplantation; NHL, non-Hodgkin lymphoma; LD, lymphodepleting; CRS, cytokine release syndrome; ICANS, immune effector cell associated neurotoxicity syndrome; ICAHT, immune effector cell-associated haematotoxicity; IEC-HS, immune effector cell-associated hemophagocytic lymphohistiocytosis-like syndrome.

In the present review we attempt to summarize the current knowledge on infectious complications occurring in patients receiving CAR-T cell therapy for lymphoid malignancies in order to provide the readers tools for better management and prevention strategies.

## Epidemiology and etiology

The real incidence of infections following CAR-T cell therapy is challenging to assess, as studies focusing on this topic are mainly retrospective and based on limited number of heterogeneous patients.

The infection incidence follows a biphasic pattern, with “early” infections rising during the first 30 days after CAR-T cells infusion, and “late” infections from day 30 onward (13–15). This classification aims to mirror the distribution of different infection-risk factors over time.

A wide range of early and late infections after anti-CD19 CAR-T in lymphoid malignancies have been observed across studies (14, 16–19), ranging from 23% to 77% and from 14% to 61% of patients, respectively. In studies regarding NHL, patients were heavily pretreated (with a median of 3.5 previous lines of chemotherapy), and up to 40% of patients had hypogammaglobulinemia at baseline. Notably, patients underwent different prophylaxis regimens before CAR-T cell therapy, mainly levofloxacin and fluconazole during neutropenia.

Similarly, Tabbara et al. reported a wide range of early infection rate of 12–50% after CD19 CAR-T therapy, and a late infection rate of 2–40% (13) across studies. Kampouri et al. recently reported a superimposable overall rate of early infections of 12–46% in patients undergoing CD19 CAR-T cells. Interestingly, although late infections were overall less common compared to the early period after CAR-T, they have been observed in up to 50% of patients 1 year after infusion (15). In this regard, Cordeiro and colleagues focused on late infections events (>90 days) after CD19 CAR-T in 54 patients who survived 1 year after therapy; 33 of patients (61%) had at least one infective event, for a total of 153 infections (18). However, most of infections were reported as mild across studies and managed in the outpatient setting. Focusing on this topic, Dizman and colleagues reported a high pooled incidence rates f or overall infection (33.8%) in a meta-analysis including more than 2000 CD19 CAR-T cell patients, but severe infections (grade ≥ 3) occurred in 16.2% of patients and the pooled estimate for infection-related mortality was low (1.8%). Though, most of the studies included did not provide data on the etiology or site of infection, or whether patients were receiving prophylaxis or not (20).

### Bacterial infections

Overall, bacterial infections prevail in the early phase after therapy, with a median time to onset of 10 days from infusion (15, 16, 21).

The incidence of microbiologically documented bacterial infections during the first month post-CAR-T ranges from 12% to 21% across studies, with a predominance of gram-positive infections (from 50% to 67%) (16, 17, 22). Gram-negative bacteremia account for a small proportion of all infections, probably mirroring the broad use of fluroquinolone prophylaxis in the CAR-T population enrolled in the trials.

In one of the largest study from Hill and colleagues, involving 133 patients who underwent CAR-T therapy with a microbiologic or histopathologic infective diagnosis, excluding possible skin contaminants, early bacterial infections occurred 24 times in 22 patients (17%); 67% of bacterial infections (16 episodes, 12% of the whole population) were microbiologically documented, with a slight predominance of gram-positive bacteremia and *C.Difficile* colitis, followed by gram-negative bacteremia with acquired or intrinsic fluoroquinolone-resistance. Notably, all neutropenic patients received fluroquinolone prophylaxis. Moreover, in the study was not specified which type of CD19 CAR-T product were administered and only 62 patients (47%) had NHL (16). Similarly, Logue and colleagues described a homogeneous population of 85 patients with NHL who received axicabtagene-ciloleucel (axi-cel), reporting an early rate of microbiologically document infection of 21% (18 episodes), the majority of whom due to *C.difficile* colitis; again, all patients received antimicrobial prophylaxis (17). These data were corroborated by a study on infection complications in a heterogeneous population of patients undergoing different type of CAR-T products in phase 1 clinical trials. Bacteremia and bacterial site infections were the most common infection type (with a slight predominance of gram positive organisms), whose rates were comparable between those who did and did not receive levofloxacin prophylaxis, casting doubt on its usefulness in these patients (23).

### Viral infections

Viral infections are the most commons in the long term period and up to 1 year after CAR-T infusion, mainly due to respiratory viruses (RV) (15). For example, a recent multicentric study on 667 patients undergoing commercial CAR-T cells (mostly anti-CD19) developing respiratory syncytial virus (RSV) infection post-infusion reported a 2-year cumulative incidence of 7%, and nearly one-third of cases (29%) progressed to pneumonia. Most infections (74%) occurred beyond day + 100 (median 8 months), with older age and profound lymphopenia being associated with severity (24). In a large analysis on more than 2000 patients undergoing CAR-T therapy (mostly CD19 CAR-T), RV infections were the most prevalent (23.3%, mainly SARS-CoV-2 and RSV) with a median onset of 160 days after infusion, whereas herpesvirus were less frequent (13.6% of patients), but occurred earlier. The most common herpesvirus infection was due to clinically significant cytomegalovirus (CMV) infections (CS-CMVi), occurring in 7.5% of cases (25).

Focusing on CMV, viral reactivation occurs in the first month after infusion, with a median time of 2–3 weeks (26). Any CMV reactivation has been reported at rates ranging from 17 to 55% after CD19 CAR-T across different studies; nevertheless, CS-CMVi occur to a lesser extent, between 3 and 15% of patients (27–31), but are associated with significant morbidity and mortality (29). CMV end-organ diseases are very rare events (affecting < 3% of patients), likely due to the use of preemptive treatments. However, CMV monitoring protocols and thresholds to start preemptive therapy varied widely across centers (32).

### Fungal infections

Invasive fungal infections (IFI) are rare events after CAR-T therapy, but potentially severe (10).

Different studies showed an overall incidence of proven/probable IFI of 2.8% in patients with NHL undergoing CD19 CAR-T cells, including 39% mold infections (mainly *Aspergillus* species), 45% yeasts infections (mostly *Candida Albicans*) and a 14% overall incidence of *Pneumocystis jirovecii* pneumonia (PJP); invasive yeast infections seem to occur early after infusion ( < 30 days), while invasive mold infections occur during both early and late phase. PJP are late events ( > 30 days) and can occur up to 1 year after infusion (33). Again, a wide range of antifungal prophylaxis were used across studies: the majority of patients received anti-yeast prophylaxis with fluconazole, while a mold-active prophylaxis was administered only in case of known risk factors (prolonged neutropenia, use of steroids or previous IFI) (16, 17, 27, 34–37).

## Risk factors

**FIGURE 1:**
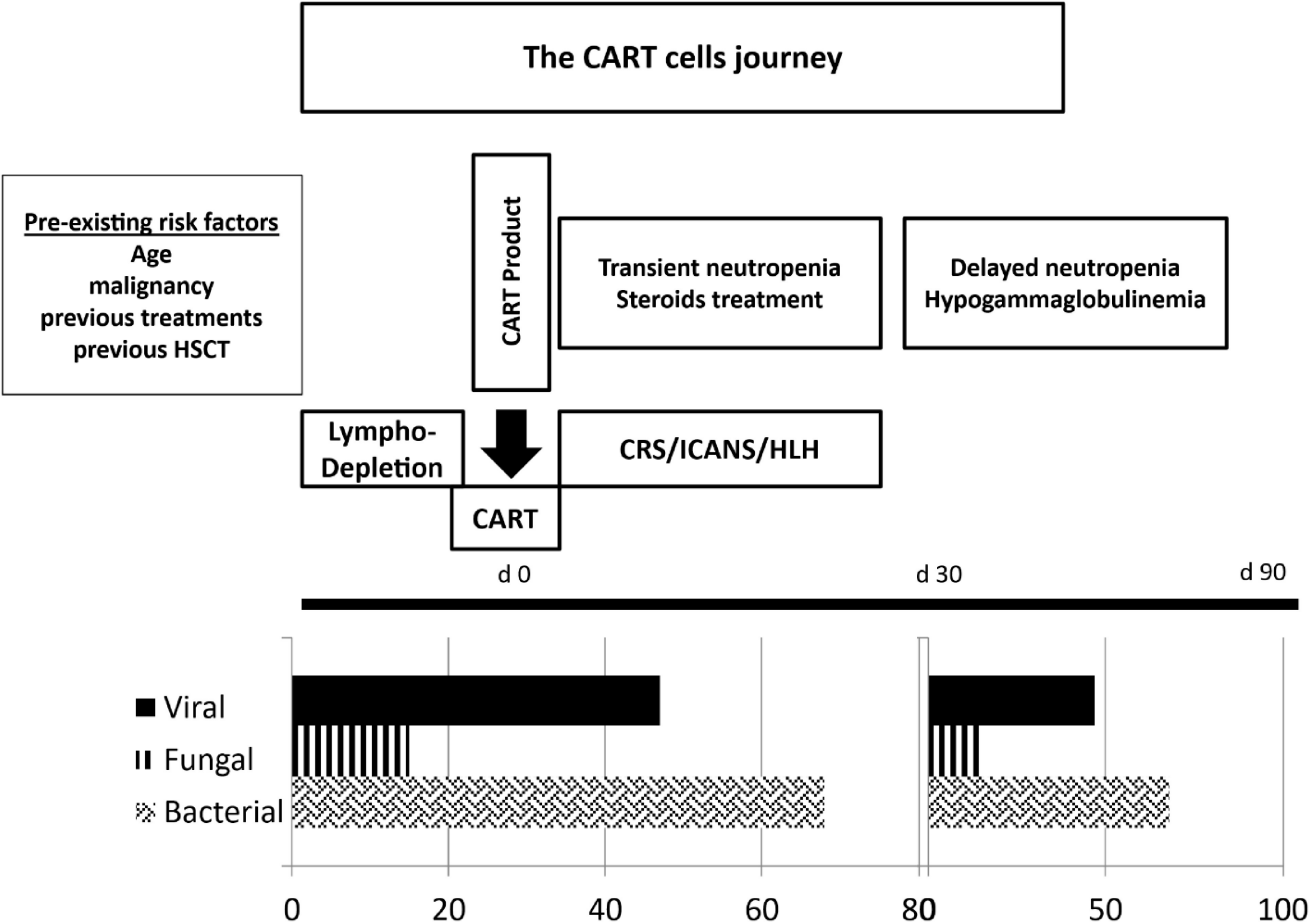
Risk factors and etiology of infections following CAR-T cells overtime. HSCT, hematopoietic stem cell transplantation; CRS, cytokine release syndrome; ICANS, immune effector cell associated neurotoxicity syndrome; HLH, hemophagocytic lymphohistiocytosis.

Risk factors associated with infectious complications in patients receiving CAR-T cell are summarized in Figure 1 and Table 1.

### Pre-infusion

Pre-infusion risk factors encompass the host immune system deficiency, resulting from the underlying malignancy, the several immuno-chemotherapy treatments and bridging therapies administered before CAR-T.

Regarding the baseline lymphoid malignancy, patients with MCL seem to have an increased risk of post infusion infections, followed by patients LBCL and FL (38–40). Indeed, a multicentric real-world study including CD19 CAR-T showed an increased rate of hematotoxicity in patients with MCL compared to those with LBCL (28% vs 23%) (41), that could increase the risk of infectious complications.

At the same time, baseline severe cytopenia [especially neutropenia with absolute neutrophil count (ANC) < 500 cell/μm^3^] related to prior therapies and/or the underlying disease correlates with a higher infection risk. In the aforementioned review, up to 35% of patients were severely neutropenic before infusion (14).

The number and type of prior therapies before CAR-T deeply affect the risk of post-infusion infections. Indeed, patients who had a prior HSCT (14, 42) or who have been heavily pretreated (16) are more likely to develop infections after CAR-T, due to the severe immunosuppression related to these conditions. For example, in a study on young adults who underwent CD19 CAR-T cells (mainly for R/R B-ALL), half of the patients received a HSCT, and experienced a remarkable increase of infections in the first 28 days after infusion (42).

Furthermore, as bispecific antibodies (BsAbs) are widely spreading in earlier treatment lines in patients with R/R LBCL and as potential bridging therapy before CAR-T cell therapy, attention has to be paid on the potential increase of infectious complications with novel immunotherapies, especially with prolonged dosing schedules over time. Indeed, a recent meta-analysis on more than 3000 patients with B-NHL showed a significantly greater infection risk (particularly grade 3 + ) for BsAbs compared to CAR-T cells (43). Similarly, Day and colleagues recently highlighted how therapies with BsAbs are burdened by a high rate of neutropenia (28–55%) and severe infections (10–40%) in R/R B-NHL (44).

Some studies investigated the impact of previous infections on the risk of infectious complications after CAR-T infusion. Mikkilineni et al. reported that recent infections ( < 100 days before CAR-T infusion) were associated with early post infusion infections in patients treated with different type of CAR-T constructs (anti-BCMA, anti-CD22 and anti-CD19) in phase 1 studies (23). Similar conclusions were reported by Wudhikarn and colleagues on 60 patients with diffuse large B-cell lymphoma (DLBCL), where the history of infections within 30 days before CAR-T cell therapy was found as a risk factor for severe bacterial infections up to 1 year after infusion (34).

### CAR-T related

CD19 CAR-T cells carry an intrinsic infectious risk, mainly due to the LD regimen and the direct effect of cellular therapy on the host immune system.

LD chemotherapy mainly consists of a combination of fludarabine and cyclophosphamide, which deprive patients of their lymphoid and myeloid compartments to allow the in-vivo expansion of CAR-T cells and improve their efficacy. Therefore, patients experience severe lymphopenia and mild to severe neutropenia in the early period after infusion (45).

Furthermore, CD19 CAR-T has “on-target, off-tumor” effects on hosts’CD19 naïve B-cells and memory B-cells compartments (46). These effects result in prolonged B-cell aplasia and hypogammaglobulinemia, which expose patients to long-term bacterial and viral infections. In this regard, a study by Baird et al. on immune reconstitution after axi-cel for DLBCL found that 60% of patients did not recover CD19 B-cells by 1 year, and that 50% of patients had a CD4 count < 200 cells/μL up to 18 months post infusion; B-cell aplasia positively correlated with the duration of CD4 T-cell reduction (35). A recent study by Riedel et al. on patients with DLBCL and multiple myeloma (MM) confirmed that CAR-T therapy leads to profound depletion of B- and T-cells, with CD4 T-cells and CD19 B-cells exhibiting impaired regeneration after treatment (47). Nevertheless, the majority of infections arise in the first 30 days after infusion, with a switch from bacterial to viral infections over time.

Lastly, the type of CAR-T construct may indirectly play a role in enhancing infectious risk with CD28 costimulatory domain-based products generally associated with higher risk in respect of 4-1BB-based compounds. For example, axi-cel showed higher efficacy, but also more toxicities (i.e., CRS) and need of steroids use compared to tisagenlecleucel (tisa-cel) in DLBCL (48). Among the pivotal studies the use of brexucabtagene autoleucel (brexu-cel) (ZUMA 2 trial) was associated with higher incidence of early grade 3–4 neutropenia (85% of patients), followed by axi-cel (ZUMA 1 trial), with neutropenia occurring in 78% of the patients (2, 9, 12, 38). Nevertheless, real-world evidence (RWE) studies have documented conflicting results in terms of different rates of infections based on the costimulatory domain associated with the CAR-T cell products (4-1BB or CD28) that may predispose patients to CRS, ICANS and ICAHT and potentially leading to a higher incidence of infections. Indeed, the use of axi-cel (CD28) is usually defined at a greater risk of CRS, ICANS and ICAHT and one study documented a higher incidence of infectious complications as compared to tisa-cel (4-1BB) (38% vs 25%, p 0.003) (49), while another one showed the opposite (tisa-cel 35.3% vs axi-cel 21.7%) (50).

### Post-infusion: early infections

The hallmark of early infections in the early phase after CAR-T infusion is leucopenia, notably neutropenia and lymphopenia, mainly due to LD therapy. Moderate to severe neutropenia develops in the majority of patients in the first days after infusion and predisposes them above all to bacterial infections. For instance, in a study on patients receiving CD19 CAR-T for both B-ALL and B-NHL, 97% developed neutropenia early after the infusion, with a median duration of severe neutropenia (ANC < 500/mcl) of 10 days (51).

CRS, the most common complication during CAR-T treatment, is a well-known risk factor for infections, in particular due to the use of high dose steroids. Furthermore, high-grade CRS has been shown to induce deeper cytopenia, more rapidly, with longer time to recovery compared to patients with a lower CRS grade (52).

In addition to steroids, the use of > 1 dose of tocilizumab to treat grade ≥ 2 CRS has been associated with an increased risk of infection in univariate analysis, but not in multivariable analysis, across different studies (16, 53, 54)). However, a study by Frigault et al. found no difference in the overall incidence of clinically significant infections or infection density in patients who received or not tocilizumab for CRS after CAR-T cells (31% tocilizumab vs 30% no tocilizumab, *p* = 0.85), suggesting that the early and limited therapy with tocilizumab used in this setting doesn’t increase the risk of infections (55).

It is now well established how CAR-T therapy can be complicated by short and long term hematological toxicity, named immune effector cell-associated hematotoxicity (ICAHT), and the cumulative risk of secondary complications, especially infections, increases with the duration of the observed cytopenia (40). The major concern is represented by neutropenia, whose duration and depth in early ICAHT has been classified in grade 1–4. In a RWE multicentric study on > 500 patients with LBCL, severe ICAHT was associated with an increased rate of severe infections and adverse treatment outcomes with higher non-relapse mortality (41).

### Post-infusion: late infections

The occurrence of late onset infections is closely associated with persistent neutropenia and lymphopenia, although the underline physiopathology of prolonged and late cytopenias is not fully understood. It has been hypothesized that marrow suppression could be due to the massive inflammatory reaction following CAR-T infusion (38), especially in patients with pre-existing decreased marrow reserve (i.e., after HSCT).

Neutropenia occurring after CAR-T infusion shows a biphasic temporal course which exposes patients to infection risk even after a first neutropenia recovery following LD. Fried et al reported that 76% of patients enrolled in his study experienced late neutropenia ( > day 21), severe in 34% of cases; also, 62% of patients developed neutropenia after > 40 days after infusion, both after resolution of previous CRS or IEC-HS (51).

As previously described, ICAHT may cause prolonged neutropenia, and ICAHT severity has an impact on the cumulative duration of neutropenia: in this respect, Rejeski et al showed how grade 4 ICAHT was associated with a median duration of severe neutropenia of 52 days (44–54), much longer than neutropenia duration observed in patients with grade 1 ICAHT (5 days) (53, 56).

As a consequence of the depletion of the B-cell compartment, CD19 CAR-T cells carry the risk of prolonged and persistent hypogammaglobulinemia, and therefore bacterial infections (34). However, in CD19 CAR-T the incidence of hypogammaglobulinemia seems to be lower compared to BCMA CAR-T, possibly because terminally differentiated B-cell have low CD19 expression levels and might survive after therapy (57).

## Predictors of hematologic toxicity

Since hematologic toxicity is commonly observed after CAR-T cell therapy and the resulting leukopenia exposes patients to a substantial infectious risk, it is crucial to identify individuals who should be considered at high risk for these complications.

Between 2021 and 2022, two retrospective analyses by Rejeski et al. involving 258 and 248 patients with R/R LBCL demonstrated that three parameters reflecting bone marrow reserve (platelet count, neutrophil count, hemoglobin) and two inflammatory markers (C-reactive protein, ferritin) strongly correlated with both the depth and duration of neutropenia, as well as with the overall risk of myelotoxicity (53, 58). These five variables, combined into the CAR-HEMATOTOX score, were shown to stratify patients into high- ( ≥ 2, HThigh) and low-risk (0–1, HTlow) categories for neutropenia and infections, particularly bacterial and severe infections. In addition, the risk of grade ≥ 3 infections was independently influenced by prolonged neutropenia ( ≥ 14 days) and extended corticosteroid exposure ( ≥ 9 days). Indirectly, high-risk patients also experienced longer hospital stays, inferior overall survival (OS) and progression-free survival (PFS).

These findings have been supported by subsequent studies in different clinical contexts (59–61).

Incorporation of baseline CAR-HEMATOTOX with procalcitonin levels, measured at the time of first fever, resulting in the HT10 score (Table 2), showed good discriminatory ability between CRS and the likelihood of an ongoing infection, particularly bacterial and severe infections, most notably in HT10 high patients (cut-off ≥ 5) (62).

**TABLE 2:** Predictive scores of hematologic toxicity.

CAR-HEMATOTOX							
Baseline features	0 Point			1 Point		2 Points	
Platelet count	> 175000/μL			75000–175000/μL		< 75000/μL	
Absolute neutrophil count	> 1200/μL			< 1200/μL		-	
Hemoglobin	> 9.0 g/dL			< 9.0 g/dL		-	
C-Reactive protein	< 3.0 mg/dL			> 3.0 mg/dL		-	
Ferritin	< 65 ng/μL			650–2000 ng/μL		> 2000 ng/μL	
Low: 0–1 High ≥ 2							
HT10							
	+ 0 Point				+ 10 Points		
Baseline CAR-HEMATOTOX	PCT ≤ 1.5 μg/L				PCT > 1.5 μg/L		
Low: 0–4 High ≥ 5							
ICAHT							
Grading	1	2			3		4
Early ICAHT (day 0–30)							
ANC ≤ 500/μL	< 7 d	7–13 d			≥ 14 d		Never > 500/μL
ANC ≤ 100/μL	–	–			≥ 7 d		≥ 14 d
Late ICAHT (after day + 30)							
ANC	≤ 1500/μL	≤1000/μL			≤ 500/μL		≤100/μL
eIPM_*pre*_			eIPM_*post*_				
Pre-lymphodepletion ANC			Pre-lymphodepletion ANC				
Pre-lymphodepletion platelet count			Pre-lymphodepletion platelet count				
Pre-lymphodepletion			Pre-lymphodepletion LDH				
Pre-lymphodepletion ferritin			Day + 3 ferritin				

ICAHT, immune effector cell-associated haematotoxicity; ANC, absolute neutrophil count.

More recently, in 2023 the European Hematology Association/European Society for Blood and Marrow Transplantation (EHA/EBMT) consensus validated the aforementioned concept of ICAHT and proposed a dedicated grading system (Table 2) based on the depth and duration of neutropenia, providing best-practice recommendations (40). These include consideration of fluoroquinolone prophylaxis in HThigh patients once the ANC falls below 500/μL. Similarly, antifungal prophylaxis should be considered in the setting of grade 3 neutropenia (according to CTCAE v5.0) and, as independent risk factors, corticosteroid administration, prior invasive aspergillosis or previous HSCT.

In this context, a model derived from the analysis of 58 variables – named eIPM (Table 2) – allows risk stratification for severe (grade 3–4) early ICAHT (within 30 days after CART infusion), and can be applied both before (eIPMpre) and after CAR-T infusion (eIPMpost) through a web-based tool^1^. eIPMPre consists of disease type (B-ALL vs. other), pre-LD absolute neutrophil count, pre-LD platelet count, pre-LD LDH, and pre-LD ferritin; eIPMPost consists of disease type (B-ALL vs. other), pre-LD ANC, pre-LD platelet count, pre-LD LDH, and day + 3 ferritin (63).

The aforementioned scores are summarized in Table 2.

## Strategies to prevent infections

Careful attention to signs and symptoms of infections is mandatory for an optimal management of patients undergoing CAR-T therapy, and strategies to mitigate infectious risk are needed. Indeed, over half of non-relapse mortality was attributed to infections in a recent extensive meta-analysis (10).

The cornerstone of infection prevention is represented by pharmacological prophylaxis; dose, timing and duration are extensively analyzed in expert recommendations and international consensus (14, 46, 64–67). However, a wide heterogeneity in infection management and prevention is reported across different centers.

The role of antibacterial prophylaxis during neutropenia after CAR-T is still a matter of debate. Some authors suggest to consider fluoroquinolone prophylaxis in patients at high risk of infection after infusion (i.e., high CAR-HEMATOTOX), but it is essential to consider local epidemiology and resistant colonization patterns, in order to avoid the further selection of multi-drug resistant pathogens (40).

Acyclovir/valacyclovir prophylaxis against HSV/VZV is recommended in all patients undergoing CAR-T cell therapy from infusion to at least 6–12 months after therapy, and/or until CD4 recovery (CD4 > 200/μL), in order to reduce viral reactivation.

The recently updated clinical practice guidelines on the use of primary antifungal prophylaxis in patients undergoing CAR-T cell (68), mainly based on the recommendations of the European Society for Blood and Marrow Transplantation (EBMT) and the American Society for Blood and Marrow Transplantation (ASBMT) (69, 70), recommend an anti-yeasts prophylaxis with fluconazole or micafungin during the neutropenia phase after CAR-T infusion (evidence B-II). Mold-active antifungal prophylaxis is needed in patients at high risk of developing IFI, namely patients with severe and prolonged neutropenia and/or those receiving high doses of steroids for the management of CAR-T complications (CRS/ICANS), and posaconazole can be considered (68). Thus, a careful evaluation of patients’ risk factors is crucial for implementing an effective prophylaxis strategy. In this respect, one study by Garner and colleagues proposed an antifungal prophylaxis protocol based on patients’ risk factors for mold infections, depending on whether they have an “AML-like” profile due to the presence of prolonged neutropenia, or a “graft-versus-host disease (GVHD)-like” profile due to the use of corticosteroids or other immunosuppressive agents, drawing evidences from the settings in which the anti-mold prophylaxis has been recommended (54, 71).

It is crucial to administer an active prophylaxis against *Pneumocystis jirovecii*, from CAR-T cell infusion to at least 6–12 months after therapy, or until the achievement of an adequate CD4 recovery (CD4 > 200/μL); the drug of choice is trimethoprim/sulfamethoxazole (pentamidine in allergic patients). It is important to consider that severe late *Pneumocystis* infections can occur, so that a high suspicion level has to be maintained after prophylaxis interruption (72).

Current practice recommendations suggest considering intravenous immunoglobulin (IVIG) supplementation in high-risk patients with IgG count persistently < 400 mg/dL and recurrent infections (64, 73), although data on the effective infection mitigation of this strategy are controversial. However, some studies showed that, despite frequent preemptive IVIG substitution, IgG levels remained persistently < 400 mg/dL in 1/4 of patients with LBCL (34, 35).

In some patients with expected high hematological toxicity (i.e., high CAR-HEMATOTOX score) or prolonged severe neutropenia, prophylactic or therapeutic granulocyte colony-stimulating factor (G-CSF) administration may be required until neutropenia recovery, in order to shorten neutropenia duration. Some concerns raised at the beginning of CAR-T practice due to a potential effect of G-CSF in inducing CAR-T toxicities (i.e., CRS and ICANS) (64), however, these initial concerns have been questioned, as early G-CSF showed to reduce the risk of febrile neutropenia with no impact on clinically relevant outcomes (40, 74). Nevertheless, no prospective trial has been conducted in this regard.

The most recent guidelines recommend CMV monitoring after CAR-T infusion only in patients who are CMV-seropositive before therapy (evidence grade AII), with a weekly active monitoring for CMV-DNAemia between 2 and 6 weeks after infusion (26). Indeed, some studies retrospectively and prospectively reported an association between CMV reactivation and increased overall mortality after CAR-T therapy in CMV-seropositive patients (30, 75).

Patients undergoing CAR-T therapy frequently exhibit attenuated vaccine responses compared to the general population and the clinical effectiveness of vaccination may be lower than in immunocompetent subjects. This finding supports the implementation of “cocooning” strategies through vaccination of household members and caregivers, as well as individualized timing of patients’ immunization based on immune reconstitution.

Current recommendations largely derive from post-HSCT vaccination programs and from emerging data on vaccine immunogenicity in CAR-T recipients (64, 76), reflecting the shared biological framework of profound therapy-induced immune impairment.

Inactivated vaccines (including influenza, pneumococcal, and hepatitis B vaccines) are generally administered from 6 months post-CAR-T, ideally in the absence of recent exogenous immunoglobulin replacement, in order to optimize vaccine immunogenicity. Live attenuated vaccines are contraindicated for at least 12 months after CAR-T infusion and until the achievement of an adequate immune reconstitution.

Regarding SARS-CoV-2 immunization, CAR-T recipients who received one or more COVID-19 vaccine doses prior to treatment should undergo a complete revaccination cycle. Revaccination should begin at least 12 weeks after CAR-T infusion and follow the full primary series and booster schedule recommended for previously unvaccinated immunocompromised patients. Although humoral responses are often reduced, booster dosing has shown to enhance immunogenicity in this population (77–80).

Whenever feasible, influenza and SARS-CoV-2 vaccination should be administered prior to CAR-T infusion, ideally at least 2 weeks before lymphodepleting chemotherapy, in order to allow a sufficient immune priming (76, 81).

## Conclusions and future directions

Although CAR-T cells have demonstrated to be effective in patients with advanced lymphoid malignancies who otherwise have a poor outcome, several unmet clinical needs persist.

Infectious complications affect a consistent number of patients receiving CAR-T cell during both the early and late phase of the procedure, with a remarkable impact on morbidity and mortality. Hematologic toxicity characterized by neutropenia and occasionally trilineage cytopenias may occur early after CAR-T infusion, but it may persist even during the late course of the treatment in at least 30% of patients. Indeed, infections and hematologic toxicity are closely connected each other as documented by several studies, and different scores have been developed to predict the likelihood of infections and cytopenias post-CAR-T. Bacteria and viruses are the predominant causative agents of infections in patients receiving CAR-T, while fungi may be detected when other concomitant risk factors (i.e., steroids) are present.

In this respect, several guidelines are available to assist clinicians in the optimal management of patients at risk of infectious complications post-CART. The indications of CAR-T cells are expanding with the inclusion of solid tumors and autoimmune diseases, and different CAR-T constructs are available: accordingly, prospective studies aiming to investigate infections in patients receiving CAR-T are of outmost importance.

Next-generation immunotherapy platforms such as bispecific antibodies and bispecific T-cell engagers (BiTEs) have been developed over the most recent years, holding the therapeutic potential in patients with several hematologic malignancies: comparative studies with CAR-T cells will define which option has the most favorable safety profile leading to a better clinical outcome.

## Footnotes

1
https://eipm.fredhutch.org/
